# Practical advice for mentoring and supporting faculty colleagues in STEM fields: Views from mentor and mentee perspectives

**DOI:** 10.1016/j.jbc.2021.101062

**Published:** 2021-08-08

**Authors:** Jennifer M. Spangle, Homa Ghalei, Anita H. Corbett

**Affiliations:** 1 Department of Radiation Oncology, Emory University School of Medicine, Atlanta, Georgia, USA; 2 Department of Biochemistry, Emory University School of Medicine, Atlanta, Georgia, USA; 3 Department of Biology, Emory University, Atlanta, Georgia, USA

**Keywords:** mentoring, collaboration, mentee, mentor

## Abstract

In 2020, the American Society of Biochemistry and Molecular Biology (ASBMB) Women in Biochemistry and Molecular Biology Committee introduced the ASBMB Leadership Awards to recognize individuals with a strong commitment to advancing the careers of women in biochemistry and molecular biology along with demonstrated excellence in research, discovery, and/or service. This innovative award recognizes efforts to mentor and support trainees and colleagues at all levels. Such a leadership award provides the opportunity to focus briefly on the important role of mentoring within the STEM disciplines. The goal of this commentary, which brings together perspectives from a senior scientist and recent recipient of the ASBMB Mid-Career Leadership Award as well as two junior faculty, is to highlight approaches for purposeful support of colleagues, with an emphasis on going beyond formal mentoring committees. The commentary primarily focuses on mentoring within the academic arena of extramural funding and publication, highlighting the reality that multiple mentors with diverse expertise and perspectives are critical to support success within STEM careers.

Much has been written about mentoring in STEM fields with entire reports and books devoted to this important topic (1, 2, 3). Indeed, this is a complex topic with many opinions and approaches that can lead to strong and supportive mentoring and many more that result in less than optimal or even damaging mentoring. Within the academic setting, most often we think about mentoring those in formal training, the students and fellows that join our research groups or programs, but equally, or potentially more critical to the culture of STEM, is the mentoring of our colleagues. Some of these colleagues are new and excited about everything that lies ahead and some may be more senior, dealing with the changing face of science and funding. Regardless of the type of mentoring, mentoring well takes time and effort, and regardless of how hard we try to do well and have a positive impact, we will make mistakes. However, when mentoring interactions work well for all involved, both parties stand to benefit and to learn from one another.

## A view from the mentoring side

As a recipient of the 2020 American Society of Biochemistry and Molecular Biology (ASBMB) Mid-career Leadership Award (Corbett), I was invited to provide my thoughts on the complex topic of mentoring in STEM. To complement my thoughts on this topic, I have asked two junior colleagues (Spangle and Ghalei) to offer advice from their perspective as current mentees in this context. My expertise on this topic comes only from years of experience, lessons learned from mistakes, and strong mentors who served as role models for me along my own academic path. When considering mentoring within an academic setting, people often think of the formal mentoring committees that are typically formed these days when a new faculty member joins a department (4). However, mentoring takes many forms outside of such committees. Some mentoring occurs in the form of informal feedback to a colleague and some in the form of collaborations. More senior faculty have the flexibility to consider how they approach such activities through a different lens than someone in an earlier career stage.

### Mentoring committees

Formal mentoring committees can be important to provide critical yet constructive feedback to new faculty on both grant and paper submissions as well as to empower them to say “no” to service requests that are not in their best interest. With respect to grant proposals, the most value typically comes from having senior faculty more familiar with the grant review process provide feedback on these proposals, particularly the research plan. Of note, providing constructive feedback often takes significant time and energy, and I have been very fortunate to have colleagues here at Emory University who have taken this charge very seriously. With this feedback in hand, the onus lies with the individual trying to consolidate the, often disparate, feedback in a cohesive manner that strengthens the proposal, which can be a major challenge.

With respect to publication, the most valuable advice the committee often gives is “focus.” For junior faculty, this advice means focus on getting one manuscript out the door. New faculty members are often excited about all the projects in their group, and this lack of focus can lead to incremental progress on all projects, rather than moving at least some work in a timely fashion to publication. As senior faculty, we can push to see the figures for the paper, which often helps to move the publication forward. We can offer to read drafts of the manuscript and to set deadlines. All of these actions may help our colleagues to get over the hump of the first publication from their independent research group, which in turn can help them to obtain their first extramural funding.

As, or more, important than the formal mentoring committee are other less tangible activities. Some of these are more relevant to individuals with less representation among STEM faculty (5, 6), which still includes women (7, 8). There is an admirable goal to have every committee assembled represent diverse voices; however, as we all know, this creates an unavoidable burden to serve for those individuals that bring those diverse perspectives to the table. As mentors, we have a responsibility to our junior colleagues to urge them to carefully consider all commitments that would distract them from what is typically their primary goal of advancing their scholarship. These decisions can relate to teaching responsibilities as well as service. Of course, these are very important areas to develop, but achieving balance is key. As mentors, one simple thing we can do is to provide the mentee with a voice that empowers them to say “No.” For example, “I consulted my mentoring committee and they have advised me to turn down this generous offer to (fill in the blank) at this point in my career.”

A flip side of providing active mentoring is the potential for “overmentoring.” Similar to overparenting (9), overmentoring comes with good intentions and stems from the heartfelt desire to ensure that junior colleagues do not fail or repeat mistakes of the past. It is important to ask yourself as a mentor how much advice is sufficient to empower a junior faculty to step into the lane without paralyzing them. A simple way to avoid this pitfall is to carefully consider the amount and impact of your advice before providing it. Ultimately, the junior colleague needs to collect the advice and make decisions for themselves and those offering the advice need to allow the space to do so. This important point of respecting the independence of a mentee to allow them the space to grow cannot be emphasized enough.

### Collaborations as mentoring opportunities

Beyond formal or informal mentoring committees, one can play a role as mentor through thoughtful collaborations. One of the most exciting aspects of asking research questions is joining forces with others who bring complementary approaches to tackle a complex question. Personally, I have benefited throughout my career from collaborations that incorporated aspects of mentoring. I have been both the mentee early in my career and I strive to be a mentor at this phase of my career. Of course, both mentor and mentee can benefit immensely from such collaborations and the roles can switch depending on who brings specific expertise to the table.

I was fortunate early in my career to meet an esteemed scientist whose world-renowned expertise in structural biology was an ideal complement to my approach primarily focused on yeast genetics. I met this colleague, mentor, and eventually life-long friend when, as a post-doc, I gave a talk at a large society meeting about the yeast mutants I had identified. He approached me following the talk to share that his group had recently solved the structure of the protein I had discussed (10) and the stars had aligned such that his group had used rational structure/function approaches to alter precisely the amino acids that my random genetic screen had identified as functionally important (11). This happenstance meeting led to a long-term collaboration that continues to this day nearly 30 years later. Early in my independent faculty career, this collaboration was fundamentally important to the success of the first grant applications from my research group and many of my initial publications (12, 13, 14, 15). My colleague remains to this day intellectually generous and highly supportive. No matter what type of statistical analysis/rigor and reproducibility I tried to apply, I could never quantitate the value this collaboration brought to my development as a scientist, as a thinker, and as a mentor.

Fast forward nearly 30 years and I strive to pay forward some of the generous mentoring that has been extended to me throughout my career by my structural biology colleague and many others. While successful collaborations depend on intangible “chemistry” that is difficult to predict or measure, I have been very fortunate to have many productive collaborations throughout my career. Several of these collaborations started when I had already navigated the tenure process to become a full professor, but my collaborators were or still are assistant professors. In considering these productive collaborations, I can appreciate several points that I perceive made them into positive experiences for me and my junior colleagues.

1) *Complementary expertise:* The collaborators bring truly complementary approaches to tackle an important scientific question. In one case, I was fortunate to recruit an excellent *Drosophila* geneticist to study RNA-binding proteins as an approach to extend my work in budding yeast to a multicellular model. This collaboration introduced me to a new model system as I had no experience with flies and my colleague to the area of RNA-binding proteins, which had not been studied very extensively in the adult fly. Similarly, I have worked closely with an outstanding biochemist and an excellent cancer biologist, both of whom have expertise in approaches and areas that I lack.
2) *Thoughtful approaches to authorship:* As I entered into several of these collaborations after becoming a full professor, I was in a position to be generous with regard to authorship. In such collaborations, where both sides bring specific expertise, the decision to be listed as co-corresponding authors is rather obvious. However, I have insisted and will continue to insist that junior members of the collaborative team have their name last on the list of authors. These scientists need the publications for their promotion portfolio while I do not. As collaborations have proceeded and my colleagues have moved through the tenure and promotion process, we have swapped the names occasionally depending on the focus of the manuscript. In addition to faculty colleagues, I have included senior members of my group as co-corresponding authors or senior authors. Generally, these individuals have been the drivers of the project, have mentored individuals in the lab, and certainly deserve this credit. Finally, the most extreme version of this mentoring through authorship choices is the offer to remove your name from the manuscript of a scientist who has moved on to become independent. This decision allows a former mentee to use a first publication that may have practically started in your group, but has been brought to fruition in their own group, as their first independent publication. Of course, there are ethical considerations for this type of decision as authorship should be decided based on measurable contributions to a manuscript (16). However, one can at least carefully consider such decisions where an additional manuscript for a senior scientist may have little impact, but that first independent manuscript can be critical to the early success of a newly independent scientist. I know many senior colleagues who generously make this decision.
One additional note I would offer from this platform is that grant reviewers have occasionally criticized me for having too many coauthor publications or publications where I am not listed as the last co-corresponding author. To address this criticism, I include my commitment to mentoring in the Personal Statement on my biosketch for all grants I submit and I mention that I try to support junior colleagues through ensuring that they get credit in the form of authorship. Perhaps grant reviewers could take this possibility, that authorship decisions may represent a commitment to supporting junior colleagues, into consideration before raising this criticism.
3) *Considering Grant Strategies:* One of the most critical goals for a new academic scientist is obtaining their first independent grant to support their research program. When we collaborate with junior colleagues, we can support their efforts to secure their first independent grant in multiple ways. Of course, we can provide advice about what mechanisms to apply for and where to apply, if NIH, what NIH institute, what study section, and then we can provide feedback on the written document, but we also need to take some practical points into consideration as part of our collaborations. One thing that is imperative is to protect the Early Stage Investigator (ESI) status for those applying for their first independent grant at NIH. As defined by NIH, ESI status applies to Program Directors/Principal Investigators who have completed their terminal research degree or end of postgraduate clinical training, whichever date is later, within the past 10 years and have not previously been awarded a substantial independent NIH research award. ESI grants with meritorious scores are prioritized for funding. There are a number of non-R01 grant opportunities that can support a collaboration without affecting ESI status of a junior member of the collaborative team. These grants are listed here: https://grants.nih.gov/policy/early-investigators/list-smaller-grants.htm. For example, a junior colleague and I recently applied for and were awarded a multi-PI R21 grant on which she is the contact MPI. The 2-year R21 Exploratory/Developmental grant is on the list of grants that do not affect ESI status. This grant benefits from our collaboration and complementary approaches while simultaneously helping my junior colleague to establish her independence without impacting her ESI status. Thus, in considering collaborations between an established and a junior faculty member, there are mechanisms for both parties to benefit from the collaboration without any negative consequences for the junior scientist.

### Help build a community and grow a network

Building a supportive network in a new environment takes time and effort, and there are many ways more established scientists can help their junior colleagues in this endeavor. Introduce junior faculty to each other and to senior faculty that they would benefit from meeting. Bring junior faculty into your network and help them integrate into communities that can support them. Help junior faculty gain visibility by referring them to study section leadership such as Scientific Review Officers (SROs), journal editors for review, or by introducing them to collaborators, visitors, or seminar speakers for scientific conversations. A recent development is the NIH Early Career Reviewer Program (https://public.csr.nih.gov/ForReviewers/BecomeAReviewer/ECR). Junior colleagues can apply for this program to participate in a study section with a small number of grants assigned as tertiary reviewer, but we can also nominate them or connect them to SROs to facilitate this activity. This window into the review process has been invaluable to several junior colleagues. As an extension of this early career experience, many journals such as JBC now also offer Early Career Reviewer (ECR) programs. The JBC has online training and the ECR program offers senior postdocs and junior faculty an excellent opportunity to learn about the review process. Scientific societies, such as ASBMB, also offer many opportunities for networking as well as the potential to forge formal mentoring relationships beyond one’s institution. All of these actions to help colleagues grow their network take minimal time and effort, but can be invaluable to the career of a junior scientist.

### Nominate colleagues for awards

This one seems obvious; however, many award nominations include a substantial amount of work for both the nominee and the nominator. Typically, there is a nomination letter, often a statement related to the topic of the award, an updated CV that is adapted to highlight the specific nature of this award, and commonly external support/recommendation letters from high profile (and often very busy) scientists are required. We have established a group of supportive female colleagues here at my institution who are proactive about nominating colleagues for awards. While I strive to nominate junior colleagues, both male and female, for awards, I have also developed a partnership with a fellow female professor where we nominate one another for awards. We have each had some success nominating one another, so we share the work of these nominations. We often also partner to nominate our junior colleagues, including through a formal committee that my colleague leads. On a practical note, it has helped to establish connections to those scientists most likely to write letters of support/recommendations for my colleague because I know who may already have a letter on file that they can quickly update to accompany a new nomination. Finally, especially for women, we should not be shy about self-nomination. Until 2020, I had never nominated myself for an award, but on the advice of several ASBMB colleagues and after much vacillating, I nominated myself for this ASBMB Leadership Award. In my self-nomination letter, I included in my rationale the goal of saving one of those ASBMB colleagues (all female scientists) the work of assembling the nomination. I urge my colleagues to not be shy about requesting nominations from others or self-nominating and, as senior scientists, we can be proactive about nominating all our colleagues for awards.

## The mentor–mentee relationship from the perspective of the mentee

### Finding the right mentor(s)

As a new assistant professor entering a vibrant research community, how does one decide which professional relationships to cultivate for mentor–mentee relationships, and, perhaps more importantly, who can be trusted to keep the best interests of the mentee at heart? While this decision is heavily nuanced and likely different for each new faculty member, below are a few considerations to keep in mind when identifying mentors.

1) *Complementary areas of expertise:* While touched on above, identifying senior faculty mentors with whom to collaborate can be especially productive when their expertise is outside of the mentee’s expertise. This approach supports the introduction of the mentee to new research circles, creates organic and mutually beneficial collaborations, and protects the research space and ideas of the mentee. This approach limits competition and builds an appreciation of each participant’s unique and critical contributions, while expanding the possibilities of research focus areas beyond what would normally be possible for the junior scientist alone.
2) *Identifying trustworthy mentors:* Identifying mentors who genuinely have the mentee’s best interests in mind can be challenging. While doing so successfully may largely be a gut feeling or somewhat of a sociological gamble, carefully considering the following points can be productive indicators: (1) Mentor allocating dedicated time from their schedule to serve in a mentoring capacity; (2) Mentor checking in periodically even when there is nothing “in it” for the mentor; (3) Mentor’s willingness to serve as a liaison to further integrate the mentee into the research community and foster new collaborations independent from existing mentor/mentee relationship; (4) Mentor’s independent acknowledgment of ESI status and actively encouraging the protection of ESI status.
3) *Mentors with shared experiences:* While some junior faculty may be fortunate to have access to outstanding mentors in-house, the options for picking the perfect mentor(s) may seem limited for others based on their location and/or interactions. One approach when seeking advice is to carefully consider all available resources. For example, #AcademicTwitter, the National Center for Faculty Development & Diversity, and various scientific Society mentorship programs can all help junior faculty to identify mentors outside their local community. There are benefits to identifying and interacting with both in-house and outside mentors that can serve as role models and whose advice resonates. Having mentors with shared experiences is invaluable. For example, as a female junior faculty with young children, career advice from a successful peer female faculty who has shared this experience is more likely to resonate than that of a male colleague who does not have children or does not share childcare responsibilities. Ideally, the academic community at large should be sufficiently diverse to offer a rich pool of trustworthy mentors to support junior faculty from all backgrounds, but this aspirational goal is still a work in progress, particularly for those from groups traditionally underrepresented in or excluded from STEM fields (6).

### Getting the most out of your mentor–mentee relationship

Much like other useful things in life that stand the test of time, nurturing a useful mentor–mentee relationship requires regular inspection and maintenance. As a mentee, setting clear short- and long-term goals with mentors and revisiting those goals routinely based on life and work updates with the mentor is critical. To keep the mentor–mentee relationship productive and lasting, communicating any changes that could impact the path to those goals to mentors is very important. Strong mentors are often as busy, or likely even busier, than junior colleagues; therefore, scheduling meetings at points in time where feedback is most valuable is key. Importantly, have an agenda or state in advance what type of specific feedback would be most helpful. For example, discussing the outcome of an unsuccessful grant submission with mentors is most useful when the summary statement has been received and digested. Similarly, discussing the specific aims of a proposal before writing the whole proposal can save time for everyone involved. While internalizing and applying all the advice received can be challenging, taking notes and keeping an open mind, considering all perspectives and then reconciling the various ideas and input following the meeting is critical. Surprisingly, the best advice is often the advice that was least appealing at the outset. In taking advice or feedback, an important point to remember is that helpful feedback is likely to be critical, but the criticism is not meant to be personal. Good mentors make this point very clear and work to provide critical yet constructive feedback.

## Conclusions/considerations/additional resources

Even a dedicated mentor does not have the time and energy to be all things to all people. Thus, the model of multiple mentors is critically important at all career stages (17). One approach is to define mentors with specific roles. While there are many avenues to identify multiple mentors, one is to work through the Thrive Mentoring Mosaic (18), which defines specific roles to mentors as Connectors, Associates, Advocates, Coaches, Mentors, and those that offer Targeted Training. For example, acting as a Connector can be the networking that introduces junior colleagues to anyone that can help them to further their career. Taking on the more traditional role of a Mentor may require more time and effort. When done well, mentoring takes time, but the rewards are significant for both the mentor and the mentee, and the best reward is when a mentor/mentee relationship evolves into a mutually beneficial relationship between colleagues. There are a number of valuable resources to build mentoring skills and connect to other individuals with a focus on mentoring. For example, the National Research Mentoring Network (NRMN) and other extensions of this network offer trainings and facilitate mentoring connections (19, 20, 21). Valuable formal training is available from the Entering Mentoring team (22, 23, 24). A combination of experience and purposeful learning about the topic can enhance mentoring skills, but in the end productive mentoring takes time and energy on the part of both the mentor and the mentee. From the outset, healthy mentoring relationships should offer benefits to both members of the mentor/mentee team and, depending on the context, both parties should learn from one another. From the senior perspective offered here, I have benefited immensely from my interactions with junior colleagues and I have certainly learned much from these individuals throughout the years. A few simple considerations, some of which are touched upon here, can aid in making such interactions positive and enriching for all involved.

## Conflict of interest

The authors have no actual or perceived conflict of interest with the contents of this article.
